# CPEM: Accurate cancer type classification based on somatic alterations using an ensemble of a random forest and a deep neural network

**DOI:** 10.1038/s41598-019-53034-3

**Published:** 2019-11-15

**Authors:** Kanggeun Lee, Hyoung-oh Jeong, Semin Lee, Won-Ki Jeong

**Affiliations:** 1 School of Electrical and Computer Engineering, UNIST, Ulsan, Republic of Korea; 2 Department of Biomedical Engineering, School of Life Sciences, UNIST, Ulsan, Republic of Korea

**Keywords:** Cancer genomics, Machine learning

## Abstract

With recent advances in DNA sequencing technologies, fast acquisition of large-scale genomic data has become commonplace. For cancer studies, in particular, there is an increasing need for the classification of cancer type based on somatic alterations detected from sequencing analyses. However, the ever-increasing size and complexity of the data make the classification task extremely challenging. In this study, we evaluate the contributions of various input features, such as mutation profiles, mutation rates, mutation spectra and signatures, and somatic copy number alterations that can be derived from genomic data, and further utilize them for accurate cancer type classification. We introduce a novel ensemble of machine learning classifiers, called *CPEM* (Cancer Predictor using an Ensemble Model), which is tested on 7,002 samples representing over 31 different cancer types collected from The Cancer Genome Atlas (TCGA) database. We first systematically examined the impact of the input features. Features known to be associated with specific cancers had relatively high importance in our initial prediction model. We further investigated various machine learning classifiers and feature selection methods to derive the ensemble-based cancer type prediction model achieving up to 84% classification accuracy in the nested 10-fold cross-validation. Finally, we narrowed down the target cancers to the six most common types and achieved up to 94% accuracy.

## Introduction

Cancer is a complex disease that refers to the phenomenon of abnormal cellular proliferation, invasion, or metastasis in human tissues and blood. It is caused by the acquisition of a series of genomic alterations. Hence, an understanding of the genetic characteristics of cancer is crucial for accurate diagnosis and treatment. Next-generation sequencing techniques have recently been widely applied to cancer research to characterize various different types of genomic alterations in cancer genomes^1–4^. Large-scale cancer genome studies have revealed that the patterns of genomic alterations are often cancer type-specific. Lawrence *et al*. found that cancer mutations vary across cancer types, with genes mutated in a cancer-specific manner^5^. Hoadley *et al*. also confirmed that tissue-specific genomic features in cancers are the dominant signals for classifying cancer subtypes^6^. In addition, somatic copy number alterations and mutation spectra also display tissue-specific patterns^7–9^.

Using the tissue-specific nature of somatic alterations in cancer, a number of prediction methods for cancer type were recently developed by employing machine learning classifiers and various mutation features to improve classification accuracy. Marquard *et al*. used a random forest classifier (i.e., one-vs-rest (OvR) binary classifiers for multiclass classification) with a feature set consisting of somatic point mutations of known cancer-associated genes, mutation frequencies, and copy number profiles from the Catalogue of Somatic Mutations in Cancer (COSMIC) database^10^ to identify tissues of origin^11^. The authors reported up to 85% maximum accuracy across six cancer types and 69% across 10 cancer types. Chen *et al*. used a support vector machine (SVM) classifier with official gene symbols, mutations, chromosome, and pathways as feature sets, which led to the maximum average accuracy of 62% across 17 cancer types^12^. Yuan *et al*. introduced a novel clustering-based feature selection scheme with a deep neural network classifier (i.e., multilayer perceptron) for cancer classification^13^. In their work, only somatic point mutations were used for input feature data, which led to the maximum accuracy of 64% across 12 cancer types. However, these previous studies have not fully assessed and exploited biologically meaningful input features and advanced machine learning techniques. Furthermore, most of them were able to handle only a small subset of the cancer types from the database for classification.

**Figure 1:**
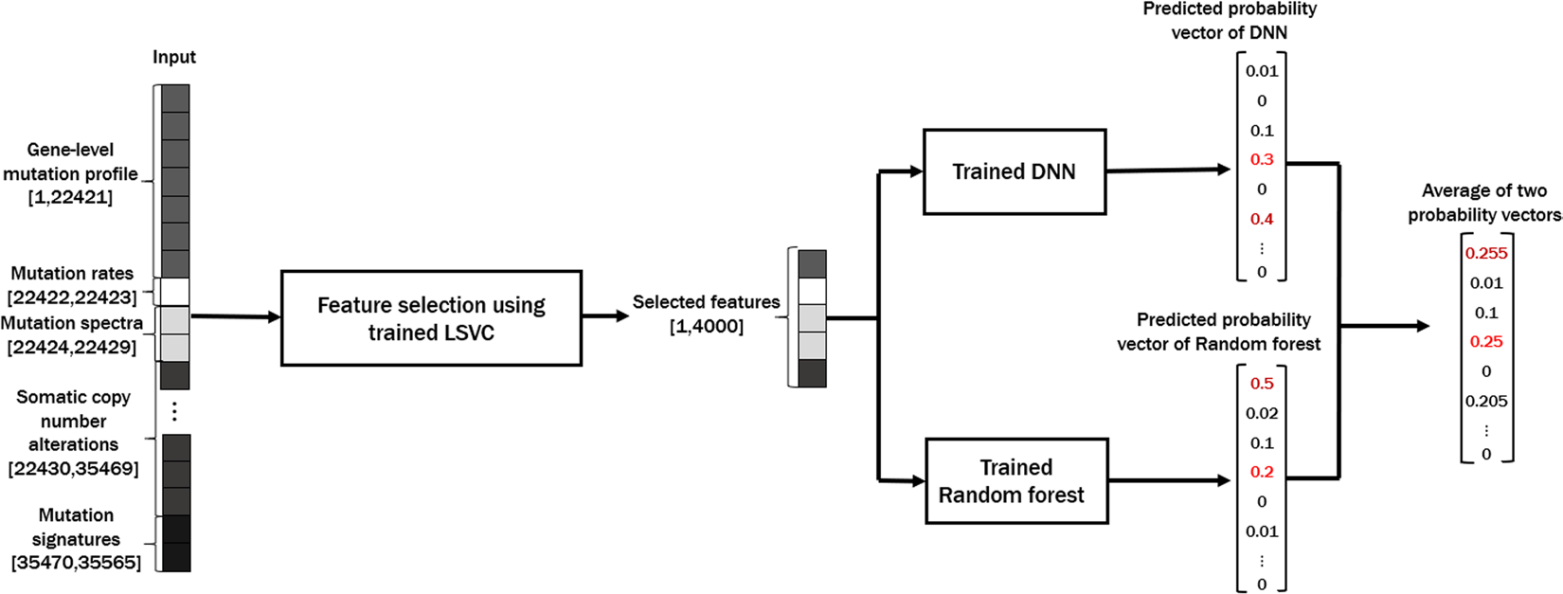
Overview of the *CPEM* cancer type prediction workflow. The first step in our workflow is building a set of feature vectors from the TCGA database. The feature vector consists of 22,421 gene-level mutation profiles, two mutation rates, six mutation spectra, 13,040 gene-level copy number alterations, and 96 mutation signatures. The next step is the feature vector dimension reduction by linear support vector classifier (LSVC)-based feature selection. In the final step, the two machine learning classifiers which were trained by the same selected feature set (a deep neural network and a random forest) are combined to build an ensemble model for the final cancer type prediction.

To address these problems, we propose a novel cancer type classification method, the Cancer Predictor using an Ensemble Model (*CPEM*) (see Fig. 1), which is based on the combination of advanced machine learning algorithms with various types of cancer somatic alterations and their derived features as input. To identify which input features are useful in classification, we conducted a comprehensive study on how various genetic alterations, such as gene-level mutation profiles, mutation rates, mutation spectra and signatures, and gene-level copy number alterations affect the accuracy of the classifier and we discuss the biological reasoning behind this result. We further investigated the performance of various state-of-the-art machine learning classifiers, both for feature selection and for cancer classification, and developed a novel cancer type classifier based on an ensemble of deep neural network (DNN) and random forest classifiers. *CPEM* can classify a large number of cancer types with high accuracy (i.e., the *average* accuracy of 84% across 31 cancer types in the nested 10-fold cross-validation). When we focused on the six most common cancer types, we were able to achieve up to 94% average accuracy. To the best of our knowledge, the proposed method is the most accurate multiclass cancer type classification method yet-devised that is able to predict the largest class of cancer types, covering the entire cancer list in the TCGA database^3^.

## Results

### Experimental setup

All experiments were conducted using a workstation equipped with an Intel Xeon CPU E5-2640 CPU with four NVIDIA GTX 1080Ti GPUs. We used TensorFlow (version 1.9.0) to implement the proposed deep neural network. Our DNN architecture is a multi-layer perceptron with a multinomial class cross-entropy with a softmax loss function. We used the Rectified Linear Unit as the activation function.

Selecting the proper number of parameters and hidden layers (i.e., tuning hyperparameters) of the DNN is important for better prediction accuracy and faster training time. We empirically chose our DNN with three hidden layers where each layer has 2048 parameters. To train the neural network, we used the Adam optimizer^14^, a commonly used stochastic optimization algorithm for machine learning, with a learning rate of 10^−5^ and 40% dropout per iteration to prevent overfitting. Other machine learning classifiers were implemented using the scikit-learn library^15^ in Python 3.6.1.

**Figure 2:**
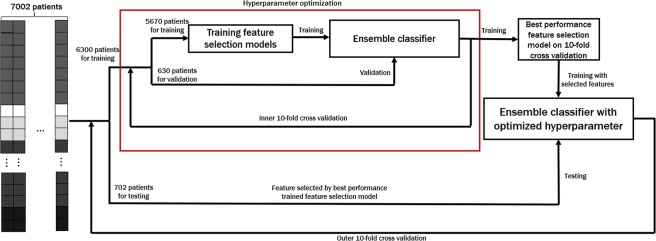
Overview of the training and testing process of *CPEM*. We employ nested 10-fold cross-validation where the optimal feature selection is performed using the inner 10-fold cross-validation while the accuracy of the proposed method is evaluated using the outer 10-fold cross-validation. In the training step, two machine learning classifiers are trained with the same selected feature set by optimal feature selection. Note that we use the testing data that is not used in the feature selection optimization step in every iteration of the outer cross-validation.

As shown in Fig. 2, the optimal number of input features and the feature selection method are chosen to maximize the accuracy over inner 10-fold cross-validation. Once feature selection is finalized, we tested the performance of *CPEM* using the independent testing data that was not a part of the training data.

### Efficacy of Various Mutation Features

**Figure 3:**
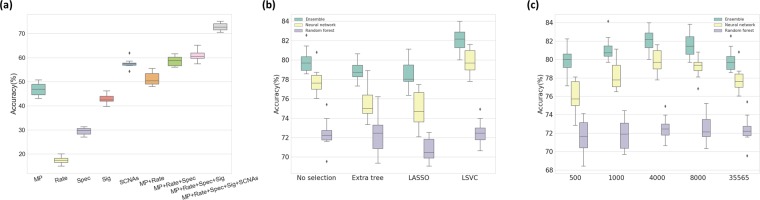
(**a**) Classification accuracy of a random forest classifier on each combination of feature groups. MP: Gene-level Mutation Profiles, Rate: Mutation Rates, Spec.: Mutation Spectra, SCNAs: Somatic Copy Number Alterations, Sig.: Mutation Signatures. (**b**) Comparison of feature selection methods. The boxplot shows the classifier accuracy measured by the inner 10-fold cross-validation for each feature selection method. It is shown that LSVC feature selection performs best on all three classifiers. (**c**) Classification accuracy for various feature sizes generated by the LSVC method. It is shown that the classification accuracy reaches its peak performance at around 4,000 to 8,000 features measured to optimize the DNN and random forest by the inner 10-fold cross-validation.

To assess how each class of input feature affected the classification accuracy, we used a random forest classifier as an initial prediction model and measured changes in classification performance as each feature was added using 10-fold cross-validation. Starting from the gene-level mutation profiles only, we consecutively added other feature groups, including mutation rates, mutation spectra, gene-level SCNAs, and mutation signatures, and measured the classification accuracy for each time. The accuracy for each feature group was 46.9%, 51.2%, 58.5%, 61.0%, and 72.7% respectively (Fig. 3a).

To further deduce the features that most contribute to the accuracy of the classification, we selected the top ten features based on the importance score from the classifier. These features included two mutation rates, one mutation signature (CCT.C > T), two mutation spectra (C > T, C > A) and five mutated genes [von Hippel-Lindau tumor suppressor (*VHL*); isocitrate dehydrogenase (NADP(+)) 1, cytosolic (*IDH1*); B-Raf proto-oncogene, serine/threonine kinase (*BRAF*); APC, WNT signaling pathway regulator (*APC*); KRAS proto-oncogene, GTPase (*KRAS*)] (Supplementary Fig. S1). This result is consistent with previous studies that identified distinct mutational landscapes from many different types of cancer genomes^8,9,16^. For example, *BRAF* is involved in intracellular signaling associated with cell growth induction and is frequently mutated in some human cancers^17^. Acquiring mutations in *KRAS* is an essential step in the pathogenesis of many cancers^18^. This result confirms that genetic features play an important role in cancer initiation and progression, and also contribute to improving the accuracy of cancer classification. The biological meaning of this result will be discussed further in the ‘Discussion’.

### Optimal Feature Selection

To choose the best feature selection method for our data, we tested the three most commonly used supervised feature selection algorithms—the extra tree-based, LASSO, and LSVM approaches. In this experiment, each feature selection method was applied to the same input feature set to reduce its size by 90% (i.e., only 10% of the input feature will be retained after selection), and the prediction accuracy of different classifiers using selected features was measured.

The accuracy of the three classifiers (i.e., ensemble, neural network, random forest) on the data processed by the three different supervised feature selection methods was compared (Fig. 3b; the inner 10-fold cross-validation results are shown using a boxplot). In general, the LASSO and LSVC selection methods performed well for the neural network classifier, and the extra tree-based selection method worked well for the random forest classifier. This is because LASSO and LSVC use the linear classifier similar to the perceptron model in the neural network, and extra tree-based selection is similar to the decision trees in a random forest. Of the LASSO and LSVC methods, LSVC displayed higher classification accuracy.

The performance of the classifier is affected by the selection method and by the number of selected features (i.e., the amount of feature dimension reduction). Figure 3c summarizes the classification accuracy for the different number of selected features, measured using the inner 10-fold cross-validation. For 35,565 features, approximately 4,000 to 8,000 selected features showed the best result, which is about 10% to 20% of the original feature set. By doing so, a higher accuracy could be achieved, up to a 2.34% increase in the outer 10-fold cross-validation, with less training time due to the small training set size.

## Putting it all Together: *CPEM* Results

**Figure 4:**
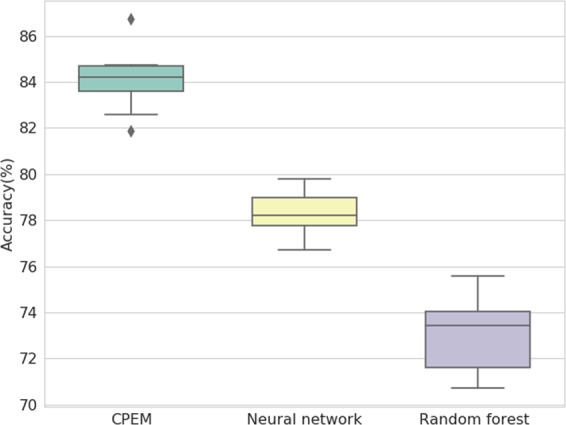
Comparison of *CPEM* and other conventional machine learning classifiers. The boxplot shows the accuracy measured by the outer 10-fold cross-validation for each machine learning model after optimization through inner 10-fold cross-validation. The average classification accuracy of *CPEM* is about 6 and 11 percentage points higher than those of a fully-connected deep neural network and a random forest, respectively.

Based on the feature selection scheme and the optimal number of features found in the previous steps, we construct the proposed ensemble-based cancer prediction model, *CPEM*. We have tested various combinations of classifiers and finally chosen a deep neural network and a random forest for our ensemble model. By combining two classifiers, we have reached up to 84% average accuracy on the testing data for 31 cancer types. This result is about 6 and 11 percentage points higher than the accuracy of conventional machine learning classifiers, such as fully-connected deep neural networks and random forests, respectively. Figure 4 shows the result of the outer 10-fold cross-validation for *CPEM* and the other classifiers, and Table 1 shows the detailed description of per-cancer type experimental result.

**Table 1:** Cancer type, sample size, number of mutated genes, number of copy number-altered genes, average precision, average recall, average F1 score and average classification accuracy of 31 cancer types used in our experiment.

Cancer type	Sample size	# of mutated genes	# of copy number altered genes	Precision (%)	Recall (%)	F1 score (%)	Accuracy (%)
Adrenocortical carcinoma (ACC)	88	6,904	12,725	96.10	84.09	89.70	84.09
Bladder urothelial carcinoma (BLCA)	127	11,674	11,223	92.04	63.78	75.35	62.20
Breast invasive carcinoma (BRCA)	967	15,716	12,178	83.19	92.14	87.44	92.86
Cervical and endocervical cancers (CESC)	191	12,165	11,505	69.63	68.06	68.84	71.89
Cholangiocarcinoma (CHOL)	35	3,511	9,707	73.68	40.00	51.85	31.43
Colorectal adenocarcinoma (COADREAD)	220	14,941	11,509	87.73	87.73	87.73	88.18
Lymphoid Neoplasm Diffuse Large B-cell Lymphoma (DLBC)	48	6,457	9,475	90.91	62.50	74.07	58.33
Esophageal carcinoma (ESCA)	184	13,557	1,1534	81.18	58.70	68.35	63.04
Glioblastoma multiforme (GBM)	280	8,069	11,410	81.85	88.57	85.08	89.64
Head and Neck squamous cell carcinoma (HNSC)	279	12,783	11,270	70.65	74.19	72.38	77.42
Kidney Chromophobe (KICH)	66	3,698	10,431	96.49	83.33	89.43	83.33
Kidney renal clear cell carcinoma (KIRC)	410	9,900	11,562	88.36	94.39	91.27	94.39
Kidney renal papillary cell carcinoma (KIRP)	161	7,332	10,568	83.23	80.12	81.65	78.88
Acute Myeloid Leukemia (LAML)	182	1,369	7,719	84.50	92.86	88.48	93.41
Brain Lower Grade Glioma (LGG)	280	4,732	10,190	89.88	82.50	86.03	82.86
Liver hepatocellular carcinoma (LIHC)	193	9,985	11,980	81.59	68.91	74.72	66.84
Lung adenocarcinoma (LUAD)	230	13,931	11,071	86.51	80.87	83.60	79.57
Lung squamous cell carcinoma (LUSC)	177	13,487	11,212	73.33	74.58	73.95	78.53
Ovarian serous cystadenocarcinoma (OV)	311	8,435	11,684	81.69	90.35	85.80	91.96
Pancreatic adenocarcinoma (PAAD)	149	10,144	8,744	83.23	89.93	86.45	89.93
Pheochromocytoma and Paraganglioma (PCPG)	166	2,125	10,103	86.78	90.96	88.82	89.16
Prostate adenocarcinoma (PRAD)	331	6,116	9,996	80.16	90.33	84.94	87.61
Sarcoma (SARC)	243	7,416	12,099	86.00	70.78	77.65	69.14
Skin Cutaneous Melanoma (SKCM)	341	17,085	11,883	90.05	92.67	91.54	92.67
Stomach adenocarcinoma (STAD)	286	16,607	11,745	76.09	79.02	77.53	80.77
Testicular Germ Cell Tumors (TGCT)	155	5,904	9,133	91.02	98.07	94.41	98.71
Thyroid carcinoma (THCA)	403	3,910	8,721	85.14	93.80	89.26	93.80
Thymoma (THYM)	121	1,805	9,305	86.14	71.90	78.38	76.03
Uterine Corpus Endometrial Carcinoma (UCEC)	242	18,412	12,133	81.39	77.69	79.49	76.86
Uterine Carcinosarcoma (UCS)	56	5,508	11,611	67.74	37.50	48.28	35.71
Uveal Melanoma (UVM)	80	1,340	9,559	80.00	85.33	82.58	83.75
Total	7,002	22,421	13,040	83.43	78.89	80.49	84.09

For better understandings of *CPEM*, we tested four well-known machine learning classifiers and further analyzed the correlation of the methods with each other. Table 2 shows the classification results of widely used classifiers, including DNN, OvR SVM, random forest, and k-nearest neighbors (KNN) clustering classifiers, in a pairwise fashion. In this table, **A** ∪ **B** is the probability that either **A** or **B** classifiers correctly predict the cancer type (total correct prediction), **A** ∩ **B** is the probability that both **A** and **B** classifiers correctly predict the cancer type (common correct prediction), and **A**−**B** and **B**−**A** are the probability that only either **A** or **B** predicts the result correctly. The classifiers were less correlated when **A** ∩ **B** was low and **A**−**B** and **B**−**A** were high. In addition, since we listed **A** and **B** in the table in descending order, it indicates that **A** is the better predictor (i.e., prediction accuracy is higher), so that **B**−**A** is the upper bound of the accuracy gain when the ensemble method is used. It is also important that the total correct prediction rate should be high (**A** ∪ **B**), and each classifier should predict higher than 50% accuracy (otherwise, it will impair the performance of the ensemble). In our experiment, we observed that the KNN clustering classifier performed worst, with only 48.59% prediction accuracy. It does not improve the accuracy when combined with other methods via ensemble. The random forest and OvR SVM performed similarly, with a respective prediction accuracy of 73.79% and 72.85%. Our DNN classifier outperformed all other classifiers, with a prediction accuracy of 82.25% which was approximately 10% higher than that of a random forest. The random forest classifier was slightly more accurate than the OvR SVM and was less correlated with DNN (i.e., lower **A** ∩ **B**). Therefore, the ensemble model combining the DNN and the random forest were the best choice. *CPEM* increased the accuracy of the DNN by 2%, which led to an average prediction accuracy of 84.09% for the 31 cancer types.

**Table 2:** Classification accuracy of various machine learning classifiers and their combinations with LSVC feature selection in the outer 10-fold cross validation.

	A	B	A ∩ B	A ∪ B	A−B	B−A	Ensemble (A, B)
DNN (A), Random forest (B)	82.25	73.79	67.81	88.23	14.44	5.98	**84.09**
DNN (A), OvR SVM (B)	82.25	72.85	70.21	84.89	12.04	2.64	80.95
DNN (A), KNN (B)	82.25	48.59	46.30	84.53	35.95	2.29	78.81
Random forest (A), OvR SVM (B)	73.79	72.85	62.58	84.06	11.21	10.27	**78.79**
Random forest (A), KNN (B)	73.79	48.59	43.87	78.51	29.92	4.71	67.30
OvR SVM (A), KNN (B)	72.85	48.59	44.50	76.94	28.35	4.08	68.41

This result confirms that the ensemble of a deep neural network and a random forest performs best.

We also compared our method to existing machine learning-based cancer type classification methods. Since it is not practically feasible to use exactly the same training data used in other studies, we collected the data of the same cancer types used in other studies from our database and compared the accuracy reported in the literature (Table 3). For the cancer types used in TumorTracer^11^, we achieved up to 91.42% accuracy for six types (85% in TumorTracer) and 90.28% accuracy for ten types (69% in TumorTracer) (Supplementary Figs S4 and S5). When using the same 12 cancer types used in DeepGene^13^, we were able to achieve up to 84.66% accuracy, which was 20 percentage points higher than that of DeepGene (64%) (Supplementary Fig. S6).

**Table 3:** Comparison between CPEM and previously reported cancer type classification methods.

	# of samples	# of features	# of cancer types	Precision	Recall	F1 score	Accuracy
DeepGene^13^	3122	1200	12	N/A	N/A	N/A	66.50
*TumorTracer^11^	2820	232	6	85.83	84.95	85.39	85.00
	4975	560	10	72.23	68.98	70.57	69.00
Chen *et al*.^12^	6751	101176	18	65.24	62.26	63.72	62.00
*CPEM	2763	4000	6	84.77	90.61	87.59	94.14
	4823	4000	14	83.48	85.36	84.41	87.02
	7002	4000	31	83.43	78.89	80.49	84.06

## Discussion

We constructed various feature groups from the collected data and confirmed a maximum of 72.2% accuracy through a combination of features using an initial prediction model based on a random forest. Feature importance was calculated to identify elements with a high impact in a combination of features with maximum accuracy. The top ten features with the highest importance were $\frac{\#\,of\,mutated\,genes}{total\,\#\,of\,genes}$, CCT.C > T mutation signature, *VHL* mutation, *IDH1* mutation, C > T mutation frequency, C > A mutation frequency, *BRAF* mutation, $\frac{\#\,of\,SNVs/indels}{1\,Mb}$, *APC* mutation, and *KRAS* mutation (Supplementary Fig. S1). The frequency of mutation varies widely among samples according to the cancer type, ranging from 0.1 (pediatric cancer) to 100 (lung cancer) per Mb. High mutation frequencies are due to extensive exposure to well-known carcinogens, such as tobacco smoke (C > A mutation) and ultraviolet radiation (C > T mutation)^9^. We referred to the COSMIC^10^ data to identify signatures with a high frequency of CCT.C > T mutations, and confirmed that the frequency of CCT.C > T mutations was the highest in Signature 23 and 19. Signature 23 is present only in liver cancer samples, and Signature 19 is specific to pilocytic astrocytoma. According to the COSMIC data, *VHL* displayed mutation rates of 39% and 33% in liver cancer and paratesticular tissues, respectively. *VHL* is a transcription factor that plays a central role in the regulation of gene expression by oxygen and is involved in ubiquitination and degradation of hypoxia-inducible-factor^19^. *IDH1* catalyzes the conversion of isocitrate to alpha-ketoglutarate (aKG) in normal conditions. The cancer-associated *IDH1* mutation converts aKG to 2-hydroxyglutarate (2HG)^20^. In gliomas and malignancies, *IDH1* mutations induce postmenopausal changes and promote tumorigenesis^21^. The frequency of *IDH1* mutations in the COSMIC data is highest in the central nervous system (34%). *BRAF* is a crucial regulator of the extracellular signal-regulated kinase – mitogen activated protein kinase signaling pathway, leading to cell proliferation, differentiation, and survival. *BRAF* mutations are present at a high frequency (8%) in various cancers, particularly in melanomas (50%)^22^. *APC* has a wide range of functions from the regulation of the *WNT* signaling pathway to cell migration, apoptosis, and chromosome segregation^23^. *APC* mutations occur in 42% of colorectal cancer and 14% of small bowel cancer. *KRAS* is a well-known oncogene that is commonly found in pancreatic, colon, and lung cancers. *KRAS* induces tumorigenesis, regulates cell degeneration, and induces genomic instability.

Even though we already achieved KG high classification average accuracy of up to 84% for 31 cancer types from the TCGA database using our ensemble method, the classification accuracy could be increased further by focusing on cancer types with a sufficient number of samples. One observation we made is that the size of the training data is not evenly distributed across the cancer types in the database. Some cancer types have a very small number of training sets, which affects the performance of machine learning (Table 1). For example, we only have 35 samples for the CHOL cancer type, which resulted in low average accuracy of 31.43%. To circumvent this issue, we selected subsets of cancer types based on the number of samples. For cancer types with 200 or more samples, we were able to collect 14 cancer types from 4,823 samples and achieved an accuracy of up to 87%, which was about 4 percent points higher than using all 31 cancer types (Supplementary Fig. S2). If we increased the threshold to 300 or more samples, then six most frequent cancer types remained (BRCA, KIRC, OV, PRAD, SKCM, and THCA), and the classification accuracy further improved to 94%, which was about 10 percentage points higher than using all 31 cancer types (Supplementary Fig. S3).

To further demonstrate the wide applicability of the proposed method to other data (i.e., non-TCGA data), we trained and tested *CPEM* using the ICGC (International Cancer Genome Consortium) dataset, which provides sequencing data for 76 cancer projects. We collected somatic SNV and CNV data of 48 cancer projects performed on at least 10 samples. In this experiment, we used the same nested 10-fold cross-validation used for TCGA data to assess the performance. *CPEM* achieved up to 82.40% accuracy, while the accuracy of neural network and random forest are 77.93% and 74.83% in outer 10-fold cross-validation, respectively. This demonstrates that *CPEM* is effective on non-TCGA data as well.

We observed that, even though CPEM demonstrates superior performance on TCGA or ICGC only, its cross-platform application is not working well, which is somehow expected in a data-driven approach. For this experiment, we collected 124 BRCA, 265 ESCA, 428 PAAD and 451 BRCA samples from the non-TCGA ICGC data. Then we directly applied our CPEM trained using only TCGA data to these non-TCGA data, which resulted in the classification accuracy of 78.23%, 6.79%, 64.95% and 49.22% for PRAD, ESCA, PAAD and BRCA, respectively. We believe the reason for the poor performance on ESCA and BRCA is due to the platform inhomogeneity. According to the ICGC portal web page (https://icgc.org/node/70708), this non-TCGA ESCA data generated by Cancer Research UK seem to also include Barrett’s esophagus as well as esophageal cancer. We believe that the histological difference between Barrett’s esophagus and esophageal cancer is significant, and this is the reason why the TCGA-trained model showed poor performance in predicting non-TCGA ESCA. As for BRCA, we compared histopathological subtypes for the tumor samples of TCGA and Non-TCGA (ICGC). Although there are overlapping major histopathological subtypes such as ‘ductal’ and ‘lobular’ carcinoma, there are histopathological subtypes that are not perfectly matched with each other. More details of BRCA’s histopathological differences can be found in Supplementary Fig. S7. Developing a more robust cancer prediction method for inhomogeneous platforms in multi-site or multi-modal data would be an interesting future research direction.

## Conclusion

We introduced a novel cancer type classification method, *CPEM* which is based on mutation features and an ensemble of machine learning classifiers. We conducted an in-depth study to clarify how various mutation feature groups affect the classification accuracy. Extensive investigation of various feature selection and classification methods based on machine learning algorithms led to the development of an ensemble model that classified 31 cancer types from the TCGA database at an average accuracy of 84% and six common cancer types at an average accuracy of 94%. The method outperforms the state-of-the-art mutation-based cancer classification methods.

Future plans are to utilize advanced deep neural networks to improve the cancer classification accuracy. Another idea we are currently exploring is to group cancer types in a hierarchical manner to reduce the search space size. We are also planning to apply our model to liquid biopsy data, such as circulating tumor DNA, and cells for primary tumor site prediction. Current liquid biopsy techniques are offered as laboratory-developed tests. Our method will be useful for early diagnosis of cancer as these liquid biopsy techniques evolve^24^. More rigorous validations using real clinical datasets will also be done.

## Methods

### Overview of the Proposed Method

CPEM consists of several data processing stages followed by a machine learning-based cancer classification stage, as shown in Fig. 1. The first step is constructing input feature vectors for various different types of somatic alterations detected from cancer genomic data to train and test machine learning classifiers. Since the input feature vectors are large and sparse (i.e., mostly zero), the next step is to reduce the size of the input feature set. This feature selection step is important to reduce the training time of the classifiers and also to increase the prediction accuracy by removing redundant and irrelevant features acting as noise. We explored several widely used feature selection algorithms^25^ and empirically chose a method that worked best for our dataset. The last step is the classification of the cancer type using machine learning classifiers. We employed an ensemble approach by combining a random forest and a deep neural network to maximize the classification accuracy. Detailed descriptions of each stage are provided in the following sections.

### Feature Construction

The genetic alterations (e.g., mutation spectra, signatures and somatic copy number alterations (SCNAs)) often have a unique pattern depending on the cancer type^8,9,26^. Hence, these characteristics are sequentially applied to the subsequent analysis. We collected somatic single nucleotide variants (SNVs) and short insertions and deletions (indels) data for 31 TCGA cancer types from the GDAC^27^ website (Jan 28, 2016). We generated a data matrix of 22,421 genes × 7,115 samples that contains the number of protein sequencing altering SNVs and indels for each gene and sample. We also downloaded SCNA data from the GDAC website and constructed gene-level log2 copy ratio values to use them as additional input features. Mutation rates and mutation spectra/signatures were calculated using SNV data to be used as input features. As a result, we generated a data matrix of 35,565 input features × 7,002 samples to develop cancer type classification machine learning models. A pictorial description of our feature matrix is shown in Fig. 1, and the details of the input feature contents are as follows:

- 22,421 gene-level somatic mutation profile
- 2 mutation rates $$(\frac{\#\,of\,SNVs/indels}{1\,Mb}\,{\rm{and}}\,\frac{\#\,of\,mutated\,genes}{total\,\#\,of\,genes})$$
- 6 mutation spectra (C > A, C > G, C > T, T > A, T > C, and T > G)
- 96 mutation signatures ({A, C, G, T} × 6 mutation spectra × {A, C, G, T})
- 13,040 gene-level log2 copy ratio

We created an initial prediction model using six machine learning models to evaluate the performance of the generated features. The initial results showed the highest accuracy in the random forest model. Subsequently, each feature was added to predict the accuracy of the initial model to verify the impact of each feature on the model.

### Feature Selection

Feature selection is the process of reducing the dimension of the input feature data by removing redundant and irrelevant features that do not contribute to the final result. By selecting important features only, the classification can be more accurate and training time can be shortened. Various feature selection algorithms for genomic data have been proposed in the past^25,28^.

In this work, we tested several widely used supervised feature selection algorithms, including tree-based, least absolute shrinkage and selection operator (LASSO), and LSVC. We finally chose LSVC as the feature selection method (the various feature selection methods are compared above in ‘Comparison of Feature selection Methods’).

#### Tree-based feature selection

The tree-based feature selection method is based on the feature importance calculated during the training of the decision tree classifier, namely how much the feature contributes to the reduction of the overall error (e.g., accuracy or variance) of the classification. Once the importance of each feature is established, the features can be sorted and those with low importance can be discarded. In our experiment, we used the extremely randomized trees (extra trees)^29^, a decision forest that avoids the expensive bootstrapping process used in the random forest algorithm.

#### LASSO feature selection

LASSO is a linear regression problem with a *L*_1_-norm sparsity term defined as follow:

1 $${\rm{\min }}\,\frac{1}{n}\mathop{\sum }\limits_{i\mathrm{=1}}^{n}\,{\Vert {y}_{i}-{w}^{T}{x}_{i}\Vert }_{2}^{2}+\alpha {\Vert w\Vert }_{1}$$

where *x*_*i*_ is the training vector, *y*_*i*_ is the predicted vector, and *w* is the regression coefficient vector.

After solving the minimization problem of Eq. 1, *w* serves as the importance value to filter the input feature *x*_*i*_. The number of selected features can be determined by the level of sparsity of *w* by changing the *α* parameter. If *w* is sparse then the small number of features will be selected, and vice versa.

#### LSVC feature selection

LSVC^30^ is based on a squared hinge loss function with an L1-norm sparsity term as follows:

2 $$\mathop{{\rm{\min }}}\limits_{{w}_{c},{b}_{c};c\mathrm{=1,...,}k}\frac{1}{n}\mathop{\sum }\limits_{i\mathrm{=1}}^{k}\,L(f,{x}_{i},{y}_{i}),$$

3 $$\,{\rm{subject}}\,{\rm{to}}\mathop{\sum }\limits_{c\mathrm{=1}}^{k}\,{\Vert {w}_{c}\Vert }_{1}\le \varepsilon $$

where *f* is a linear decision function defined as *f*_*c*_(*x*) = *w*_*c*_^*T*^*x* + *b*_*c*_ for each class *c* = 1,..., *k* in *k*-class. In our experiment, we choose the *f* as one-vs-rest decision function for multi-class classification. The loss function *L* is defined as follows:

4 $$L(f,{x}_{i},{y}_{i})=\sum _{c\ne {y}_{i}}\,{\rm{\max }}\,{\mathrm{(0,1}+{f}_{c}({x}_{i})-{f}_{{y}_{i}}({x}_{i}))}^{2}$$

Since *w* for each class will be sparse after minimization, important features can be selected by collecting elements with non-zero *w*. Similar to LASSO, the total number of selected features is determined by the sparsity of *w*, which can be controlled by changing *ε*.

### Cancer Type Classification

Once feature data are collected and processed, the next step is training machine learning classifiers to predict cancer types. Most previous studies used a collection of binary classifiers (i.e., one-vs-rest)^11,12,31^ to predict the cancer types. Popular classifiers are random forests and support vector machine, with deep neural networks used most recently^13^. Rather than relying on one specific classifier, as in the previous studies, we proposed to build an ensemble of different machine learning methods, which is a widely used strategy to enhance the classification performance^32,33^. To achieve this, we tested four widely used machine learning classifiers, including random forests, OvR SVM, KNN, and fully connected DNN, and found the best classifiers for our ensemble model. To build an ensemble, we used multinomial classifiers rather than binary classifiers, and computed the average of the probability of each output to determine the per-label (i.e., cancer type) probability. We performed an in-depth analysis of the correlation of output from the classifiers (see ‘Putting it all together: *CPEM* results’ in the Results).

## Supplementary information

Supplement file

## Data Availability

The datasets and code used in this study are available on https://github.com/leekanggeun/CPEM.
