# Supplementary material for: CPEM: Accurate cancer type classification based on somatic alterations using an ensemble of a random forest and a deep neural network
Source: Sci Rep. 2019 Nov 15;9:16927. doi: 10.1038/s41598-019-53034-3 (PMC6858312; doi:10.1038/s41598-019-53034-3)
Supplement: Supplementary file 1 — Supplement file [file 41598_2019_53034_MOESM1_ESM.pdf]

CPEM: Accurate cancer type classification based on somatic alterations using an ensemble of random forests and a deep neural network

Kang-geun Lee[1,†], Hyoung-oh Jeong[2,†], Semin Lee[2,*] ,and Won-Ki Jeong[1,*]

[1]School of Electrical and Computer Engineering, UNIST, Ulsan, 44919, Republic of Korea.

[2]Department of Biomedical Engineering, School of Life Sciences, UNIST, Ulsan, 44919, Republic of Korea.

Supplementary Material

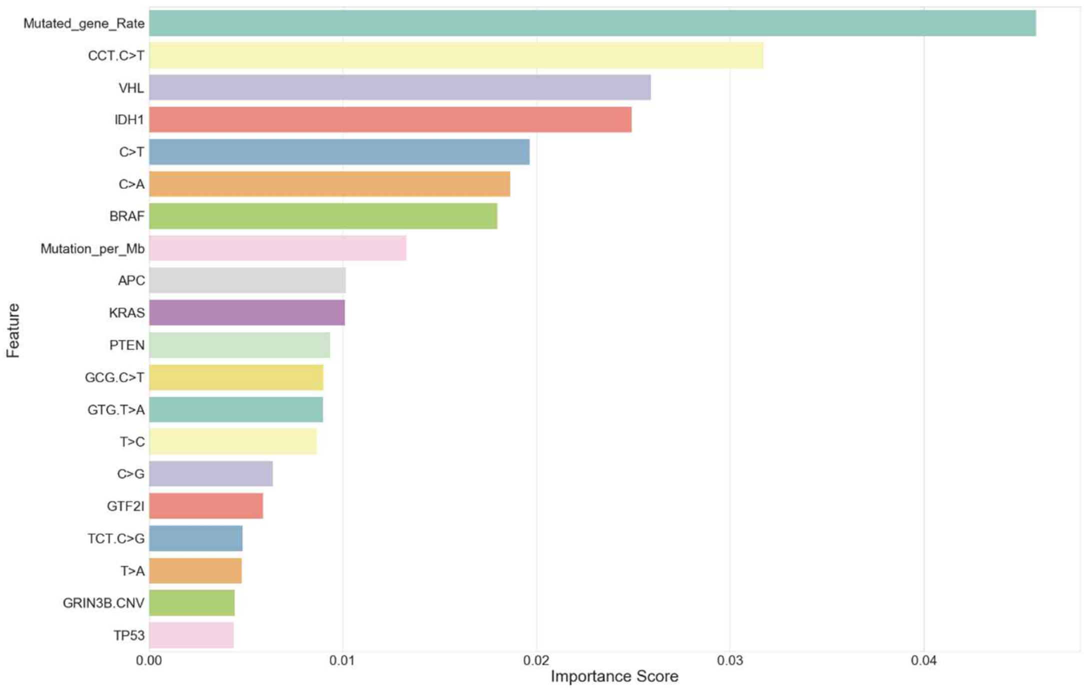

Figure S1: This figure is a bar graph that sorts as the importance for each feature in “3.2. Efficacy of Various Mutation Features”.

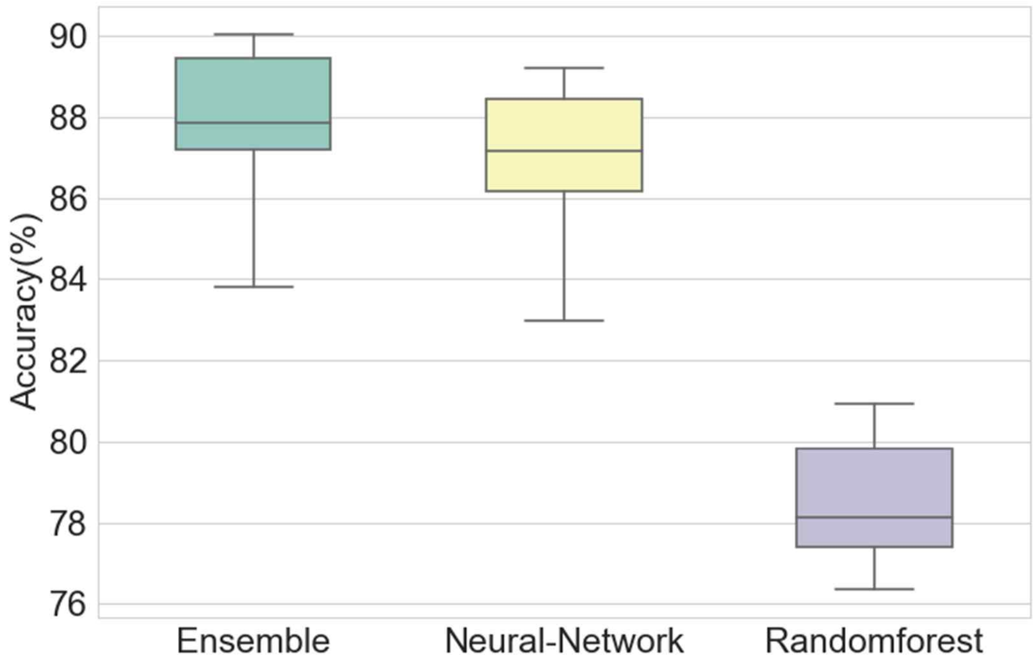

Figure S2: This boxplot is the result of Ensemble, Neural Network and Random forest with only classes with more than 200 samples.

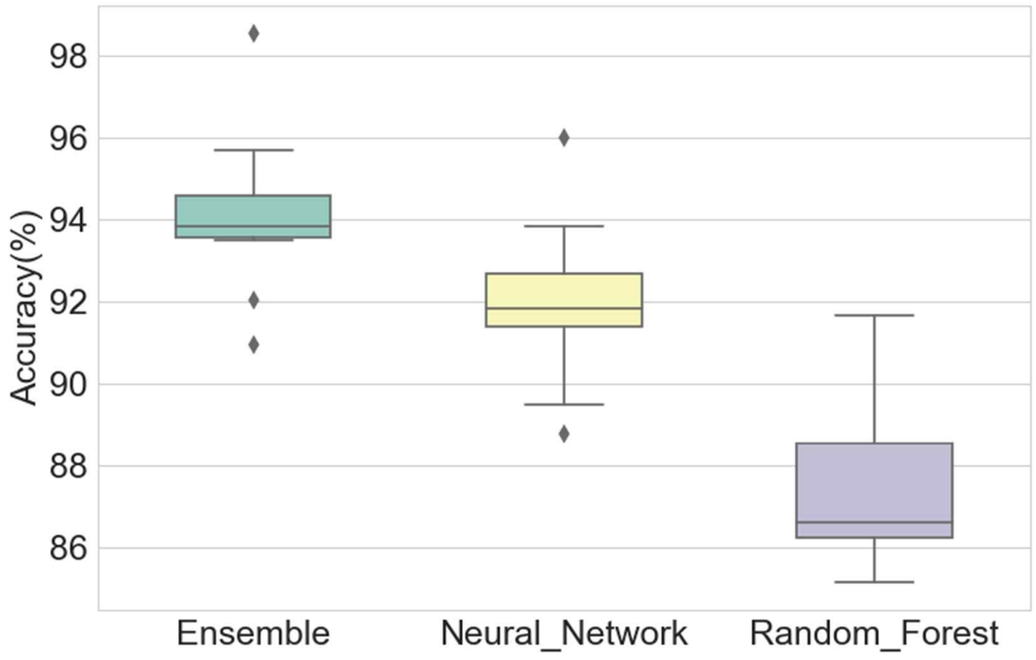

Figure S3: This boxplot is the result of Ensemble, Neural Network and Random forest with only classes with more than 300 samples

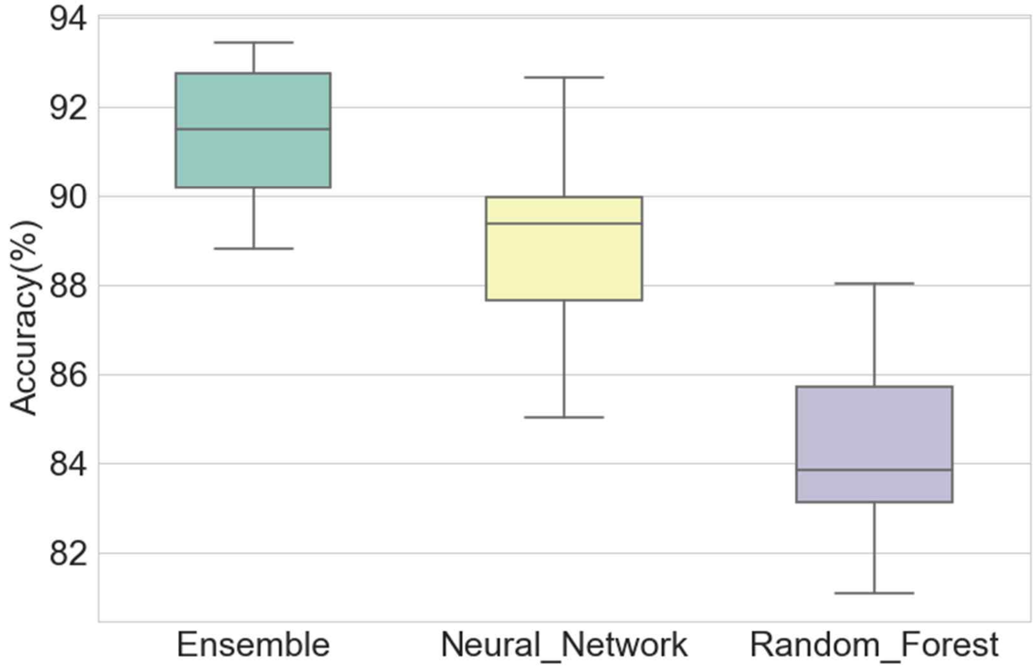

Figure S4: This boxplot is the result of Ensemble, Neural Network and Random forest with only classes the 6 cancer types used in TumorTracer .

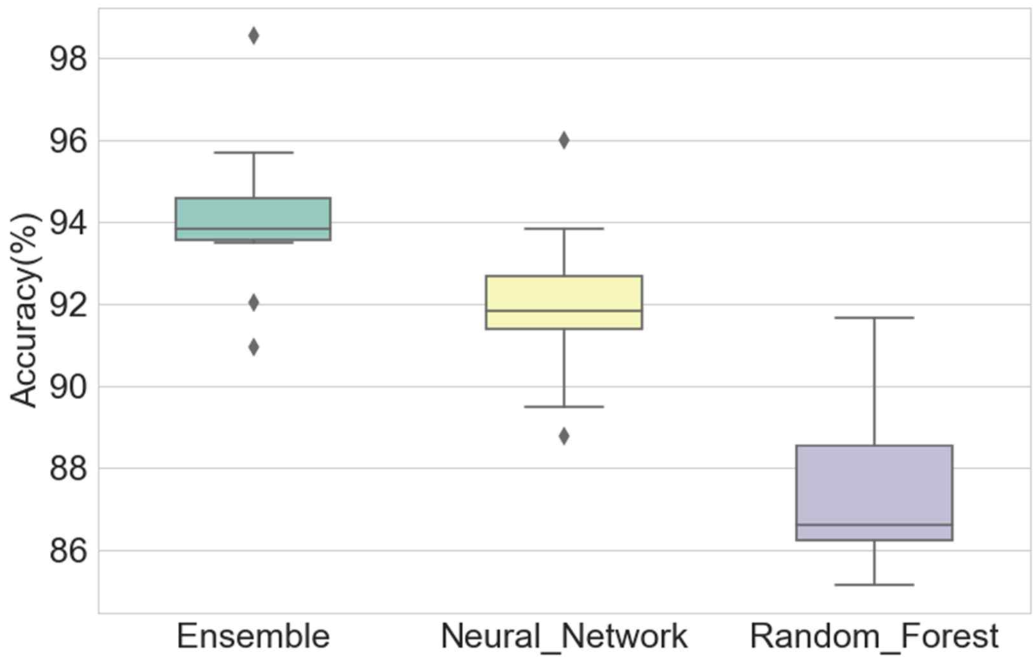

Figure S5: This boxplot is the result of Ensemble, Neural Network and Random forest with only classes the 10 cancer types used in TumorTracer .

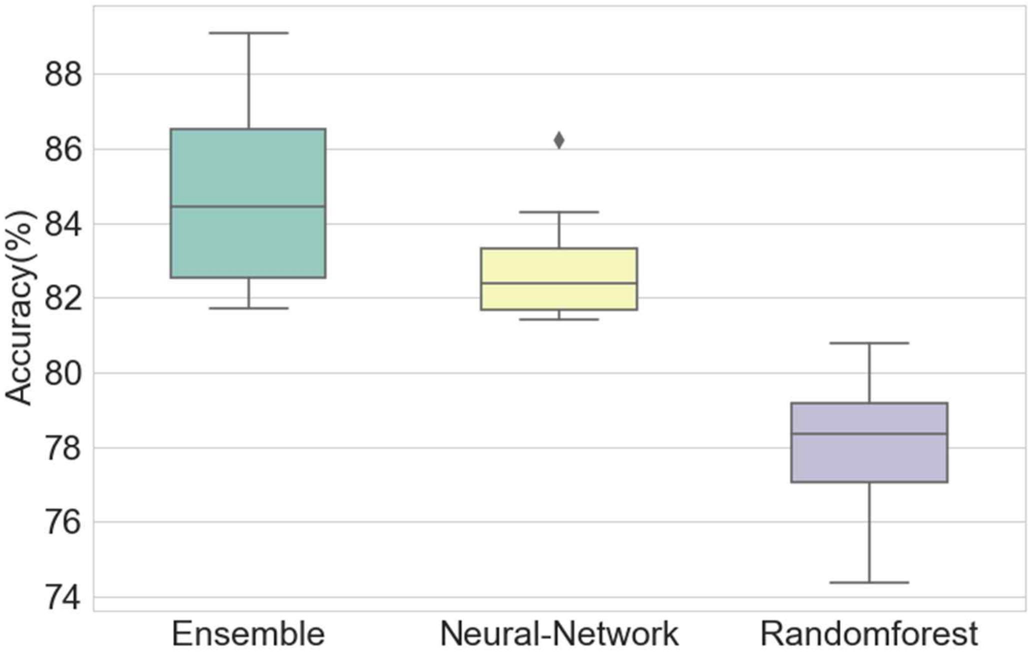

Figure S6: This boxplot is the result of Ensemble, Neural Network and Random forest with only classes the 12 cancer types used in DeepGene.

TCGA	ICGC (non-TCGA)
infiltrating carcinoma nos	adenoid_cystic_carcinoma
infiltrating ductal carcinoma	apocrine
infiltrating lobular carcinoma	cribriform_tubular
medullary carcinoma	ductal
metaplastic carcinoma	lobular
mixed histology	lobular_Pleomorphic
mucinous carcinoma	metaplastic
other, specify	micropapillary
	mucinous
	neuroendocrine
	no_data_supplied
	papillary

Figure S7: Histopathological subtypes of breast cancer samples from TCGA and ICGC(non-TCGA)
